# Editorial: Advances in novel drugs and targets for hepatic and gastrointestinal diseases

**DOI:** 10.3389/fphar.2024.1364290

**Published:** 2024-01-15

**Authors:** Ebenezeri Erasto Ngowi, Yong Gao, Xiaoying Yang, Aijun Qiao

**Affiliations:** ^1^ Zhongshan Institute for Drug Discovery, Shanghai Institute of Materia Medica, Chinese Academy of Sciences, Zhongshan, Guangdong Province, China; ^2^ Shanghai Institute of Materia Medica, Chinese Academy of Sciences, Shanghai, China; ^3^ University of Chinese Academy of Sciences, Beijing, China; ^4^ Science and Technology Innovation Center, Guangzhou University of Chinese Medicine, Guangzhou, China; ^5^ Jiangsu Key Laboratory of Immunity and Metabolism, Xuzhou Medical University, Xuzhou, China

**Keywords:** novel drugs, target, liver diseases, gastrointestinal diseases, molecular mechanism

## Introduction

The escalating global incidence of gastrointestinal and hepatic diseases, encompassing both malignant and non-neoplastic conditions, presents significant health and economic burdens. Despite the progress made in understanding these diseases and developing treatments, the exact mechanisms remain unclear, and targeted medications are yet to be developed. The urgency to discover novel treatments for these diseases is paramount. As a result, this Research Topic centers on novel targets and potential therapeutic strategies, along with the underlying mechanisms which may contribute to the pathogenesis of hepatic and gastrointestinal diseases. This Research Topic brings together a collection of 32 papers, comprising 17 research articles, 11 review articles, 3 clinical trials, and 1 study protocol. Finally, we extend our deepest gratitude to the authors, reviewers, and the diligent editorial team for their steadfast dedication and commitment to advancing knowledge in this vital domain.

## Hepatic diseases

Hepatic diseases represent a range of disorders that impact the liver. These can be either inherited or triggered by complex factors like viruses, alcohol, metabolic diseases, and certain medications. If left untreated, these diseases can progress to serious complications, including liver failure. In this Research Topic, advances made in understanding and treating liver diseases have been reported. An in-depth review by Yuan et al. describes the origin and characteristics of tumor-associated macrophages and their role in hepatocellular carcinoma (HCC) formation. Meanwhile, review articles provided by Shi et al. and Li et al. exhaustively discuss the use of traditional medicines and natural supplements for treatment of liver diseases. The advancement of liver diseases may necessitate a liver transplant, a procedure that is not without its own set of diverse complications. To this end, Jia et al. performed a meta-analysis of nine clinical trials to determine whether dexmedetomidine (DEX) is able to improve liver transplantation results, and found that DEX could enhance clinical outcomes and shorten hospital stays. The authors concluded by advocating for further research to ascertain its effectiveness and potential side effects.

## Hepatitis B and C

Hepatitis B and C are prevalent viral infections that result in liver inflammation and are primarily transmitted via bodily fluids and blood, respectively. Both infections can progress to chronic liver diseases. Tenofovir amibufenamide (TMF) and tenofovir alafenamide (TAF) are both identified as primary treatments for chronic hepatitis B (CHB). TMF is structurally distinguished by an additional methyl group, which enhances its liposolubility and, consequently, its activity. Li et al. compared the clinical efficacy and safety of TMF and TAF in CHB patients. They uncovered that TMF is more effective and safer than TAF at deterring viral replication, and without noticeable side effects in renal activity and blood lipids. On the other end, Fang et al. conducted a nationwide retrospective cohort analysis on the risk of neuropsychological disorders (NPDs) in chronic hepatitis C (CHC) patients who received either interferon (IFN) therapy or direct-acting antivirals (DAA) therapy. They found that treatment-naïve CHC patients receiving DAA therapy had a lower incidence of NPDs compared to those receiving IFN therapy, but the difference was not observed in the retreatment DAA group.

## Non-alcoholic fatty acid liver diseases

Non-alcoholic Fatty Acid Liver Disease (NAFLD), is the most prevalent liver disorder, marked by an over-accumulation of fat in hepatocytes and is highly associated with complex diseases such as obesity, type-2 diabetes mellitus (T2DM), and cancer. The accumulated evidences have shown that the occurrence of those diseases partially attribute to the dysregulation of the immune system, which is profoundly impacted by the interplay between gut microbiota and dietary factors. A study by Huang et al. explored the potential of plant extract, inulin, in alleviating metabolic syndrome using mice model. They demonstrated that inulin has the capacity to improve glucose and lipid metabolism traits in mice by altering the gut microbiota, thus elevating bile acid excretion and reducing intestinal lipid absorption. In another study, Ma et al. found that sulfosuccinimidyl oleate can improve insulin resistance and lipid metabolism in high fat diet-induced obese mice. In addition, Kang et al. conducted a study on the efficacy of Cheong-sang-bang-pung-san extract (CB) for improving liver steatosis and its underlying mechanisms. They observed that CB treatment can effectively lower lipid accumulation in the liver of HFD-fed mice. Furthermore, by using free fatty acid-exposed HepG2 cells, the authors were able to associate CB effect with the stimulation of AMP-activated protein kinase activity. With regard to NAFLD treatment, several contributions have been made. A systematic review by Wang et al. concluded that probiotics are most likely to improve NAFLD markers compared to other interventions, and professional diet and exercise advice outperformed no intervention. Similarly, a network meta-analysis conducted by Jin et al. evaluated the effectiveness of current anti-diabetic drugs, specifically rosiglitazone and vildagliptin, in treating NAFLD in non-diabetic patients. Their findings indicated that rosiglitazone and vildagliptin are most effective for improving alanine aminotransferase and aspartate aminotransferase, respectively. Studies from Zhu et al. and Shiragannavar et al. provided fascinating insights into the potential of natural remedies, arecoline and withaferin A, respectively, for the treatment of NAFLD. The two compounds are efficient in lowering lipid accumulation and improving related disease parameters. A randomized controlled trial by Tutunchi et al. involved a 12-week treatment with oleoylethanolamide (OEA) and a low-calorie diet in sixty obese and NAFLD patients. The results revealed a significant improvement in oxidative stress and antioxidant biomarkers, although no notable changes were observed in inflammatory indicators. Yang et al. provided insight into the role of N-acetylcysteine (NAC) in the treatment of NAFLD and associated mechanisms. The authors reported that NAC could potentially improve inflammation-induced liver damage, hepatic steatosis, and glucose intolerance by replenishing hepatic glutathione (GSH) and GSH reductase levels in animals with NAFLD. Collectively, these studies provide a solid platform for studying these medicines’ mechanisms and clinical efficacy in NAFLD.

## Liver damage

Sepsis and the excessive intake of certain medications, such as acetaminophen, can each lead to liver damage, through various inflammatory responses and the generation of detrimental metabolites, respectively. This Research Topic explores compounds that exhibit potential in mitigating liver damage instigated by sepsis and the overconsumption of acetaminophen. Zhao et al. investigated the effect of artemisitene on ferroptosis in sepsis-induced liver damage and associated mechanisms. They found that artemisitene can significantly attenuate liver damage and ferroptotic events by downregulating nuclear factor kappa B (NF-κB) and upregulating the nuclear factor erythroid 2-related factor 2/heme oxygenase 1/glutathione peroxidase 4 pathways. In addition, Shaker et al. examined the impact of alpelisib, a phosphatidylinositol 3-kinase alpha inhibitor, on acetaminophen-induced liver damage. They discovered that alpelisib, can effectively alleviate both inflammation and immune cell infiltration caused by acetaminophen.

## Liver fibrosis

Liver fibrosis is a chronic disease characterized by excessive scar tissue in the liver. The condition is profoundly linked to ferroptosis, a unique form of iron-dependent cell death. A research contribution by Xu et al. found that doxofylline, a bronchodilator, exhibited an anti-fibrotic effect in mice with CCL4-induced liver fibrosis. Their results also revealed that doxofylline effectively reduces liver fibrosis markers via suppression of transforming growth factor beta (TGF-β)/Smad signaling pathway and inducing ferroptosis in hepatic stellate cells.

## Gastrointestinal diseases

Gastrointestinal diseases encompass a spectrum of conditions, from minor disturbances to severe functional and structural abnormalities, all impacting the digestive system and significantly affecting an individual’s quality of life. An excessive growth of bacteria in the small intestine is indicative of a condition known as small intestinal bacterial overgrowth (SIBO), which can lead to a variety of gastrointestinal complications, including diarrhea. A study protocol reported by Guo et al. describes a prospective, single-center, open-label, double-arm randomized controlled trial designed to evaluate the relative effectiveness of berberine, a natural compound present in several medicinal plants, and rifaximin, a widely used antibiotic, in the treatment of patients diagnosed with SIBO. Furthermore, Mady et al. employed UHPLC/MS profiling and *in vivo* techniques to examine the phenolic content of 80% aqueous methanol extracts of *Q. robur* and *Q. coccinea* levaes and to evaluate their subsequent anti-diarrheal effects in mice. The authors associated the resultant antidiarrheal effects with the inhibition of gastrointestinal activity and secretion by flavonoids and tannins in the leaf extracts.

## Functional dyspepsia

Persistent discomfort in the stomach, marked by symptoms like upper abdominal pain, bloating, and nausea, are key features of functional dyspepsia (FD). In this Research Topic, two articles focused on the potential of traditional medicines for managing FD. A systematic review by Kim et al. thoroughly examined the use of a traditional Chinese medicine, *Banxia-xiexin tang* (BXT), as a potential treatment for FD. Their analysis, which compared BXT to Western treatments and evaluated its potential as a complementary therapy, concluded that BXT is both safe and effective. Ha et al. conducted a comprehensive, multi-center, double-blind, placebo-controlled study to assess the effectiveness of Naesohwajung-tang, a traditional Korean herbal blend, in treating FD patients.

## Inflammatory bowel diseases

Inflammatory Bowel Disease (IBD) is a prevalent disorder characterized by chronic inflammation in the digestive tract. Patients with IBD often undergo bowel preparations prior to procedures like colonoscopies. However, it is important to note that certain medications, particularly those targeting the gastrointestinal system, can potentially interfere with these preparations, resulting in insufficient bowel cleansing. A study by Tong et al. investigated the impact of liraglutide, a glucagon-like peptide-1 receptor agonist, and sitagliptin, a dipeptidyl peptidase-4 inhibitor on bowel preparation and gastrointestinal symptoms in T2DM patients. The authors found no evidence of increased gastrointestinal complications risk linked to either treatment in T2DM patients. However, in patients with diabetic peripheral neuropathy, they discovered that liraglutide could potentially increase the risk of insufficient bowel preparation. Jang et al. examined the potential of sinapic acid, a plant extract, in treating IBD. Their results indicated that the compound can improve aberrant intestinal permeability, imbalanced gut microbiota, and inflammation potentially through its direct binding to TGF-β-activated kinase 1, leading to changes of its downstream targets.

## Pancreatitis

Pancreatitis encompasses acute to chronic inflammation of the pancreas, which is a crucial organ for digestion and blood sugar regulation. Specific and efficient therapeutic options for managing pancreatitis are still lacking. In their systematic review, He et al. examined numerous randomized clinical trials to assess the efficacy of an anti-cholinesterase drug, neostigmine, in treating patients with non-mild acute pancreatitis. They ascertained that the administration of neostigmine aids in the restoration of gastrointestinal function and could potentially enhance the overall clinical outcome. Huangfu et al. presented preliminary data to support the improvement of severe acute pancreatitis following xanthohumol treatment in mice. They revealed that xanthohumol mitigates oxidative stress and improves autophagy via inhibition of AKT/mTOR signaling.

## Gastrointestinal carcinoma

Gastrointestinal carcinomas in general, are characterized by a multifaceted etiology and are addressed through a diverse array of management approaches. Recent advancements have led to a significant progress in understanding the molecular mechanisms of the disease, developing innovative treatments, and improving methods for early detection. In this Research Topic, Zhou et al. provided a comprehensive review, evaluating treatment trends for gallbladder cancer and highlighting both current and future prospects. Tumor-infiltrating lymphocytes (TILs) play crucial role in development and progression of cancer. With regard to gastric cancer, Zhou et al. explored the role of calponin1 in the increasing angiogenesis and inflammation properties. The authors uncovered a distinctive correlation between the irregular expression of calponin1 and the marker genes of TILs, particularly tumor necrosis factor receptor superfamily member 14 and neuropilin 1.

Moreover, several studies within the scope of this Research Topic have specifically concentrated on colorectal carcinoma (CRC). Within this framework, both Huang et al. and Li et al. delivered comprehensive reviews on the latest progress in targeted therapies and their pathways, clinical studies, and the implementation of precision medicine in CRC treatment. With regard to natural compounds, Brockmueller et al. conducted a study on the impact of the turmeric-derived anticancer compound, calebin A, on the regulation of cell migration in CRC treatment. In their study, they associated the observed anticancer effect with the partial inhibition of the hypoxia-inducible factor-1α/NF-κB pathway. Under certain circumstances, a patient could concurrently or successively develop both CRC and HCC. Here, an in-depth analysis of microarray data to explore the molecular mechanisms of CRC when it is complicated with HCC was conducted by Gao et al. They identified a multitude of genes that are differentially expressed in both HCC and CRC. This research laid a vital foundation for further studies aiming to explore the mechanism associated with the complication.

## Summary

We are pleased to present this Research Topic in the hopes that it will help further our understanding of the new molecular processes of pathogenesis, possible treatment agents, and pharmacological impacts of gastrointestinal and liver diseases. This Research Topic has well-presented research articles covering a broad variety of gastrointestinal and liver ailments. Furthermore, there has been a plethora of research into the efficacy of natural therapies and traditional medicinal practices. As additional research continues, we believe more innovative findings and breakthroughs will be available for the clinical application of hepatic and gastrointestinal diseases. Finally, although several treatments have been developed, their exact molecular mechanisms and efficacy still need further investigation.

